# Visit-to-Visit Fasting Glucose Variability in Young Adulthood and Cardiac Structure and Function at Midlife: The CARDIA Study

**DOI:** 10.3389/fcvm.2021.687054

**Published:** 2021-09-16

**Authors:** Zhenyu Xiong, Peihan Xie, Jiaying Li, Zhi-chong Chen, Yifen Lin, Menghui Liu, Shaozhao Zhang, Xiangbin Zhong, Huimin Zhou, Xiaodong Zhuang, Xinxue Liao

**Affiliations:** ^1^Department of Cardiology, The First Affiliated Hospital, Sun Yat-sen University, Guangzhou, China; ^2^Key Laboratory on Assisted Circulation, Ministry of Health, Guangzhou, China; ^3^Department of Ultrasound, The First Affiliated Hospital, Sun Yat-sen University, Guangzhou, China; ^4^Guangdong Provincial Geriatrics Institute, Guangdong Provincial People's Hospital, Guangdong Academy of Medical Sciences, Guangzhou, China; ^5^Cardiovascular Department, The Sixth Affiliated Hospital of Sun Yat-sen University, Guangzhou, China; ^6^Center for Information Technology and Statistics, The First Affiliated Hospital, Sun Yat-sen University, Guangzhou, China

**Keywords:** fasting glucose variability, echocardiography, left ventricular structure and function, young adults, long-term follow-up

## Abstract

Glycemic variability was found associated with left ventricular structure and function in type 2 diabetes. But it is still unclear that whether the greater visit-to-visit fasting glucose (FG) variability in young adulthood among the community population is associated with cardiac function alteration and cardiac remodeling at midlife. The community-based prospective cohort study of Coronary Artery Risk in Young Adult (CARDIA) recruited young participants at the baseline age of 18–30 years during the period of 1985–1986 (Year 0). FG was measured at Year 0, 2, 10, 15, 20, and 25. The echocardiographic evaluation of cardiac structure and function was conducted at year 25. A total of 2,600 young adults mean (SD) aged at 24.9 years (3.6) of which 57.3% were women and 46.7% were African Americans had been included in the study. After multivariable adjusted, higher SD of mean FG (SD_FG_) is associated with lower early peak diastolic septal mitral annular velocity (e') (β [SE], −0.214 [0.080], *P* < 0.01) and higher E/e' (β [SE], 0.307 [0.094], *P* < 0.01), and higher coefficient of variation of the mean FG (CV_FG_) is also associated with lower e' (β [SE], −0.141[0.066], *P* < 0.05) and higher E/e' (β [SE], 0.204 [0.078], *P* < 0.01). The higher average real variation of mean FG (ARV_FG_) is associated with higher E/e' (β [SE], 0.178 [0.085], *P* < 0.05) and higher left ventricular mass index (LVMI) (β [SE], 1.240 [0.618], *P* < 0.05). The higher FG variability in young adulthood is associated with the subclinical change of left ventricular (LV) diastolic function at midlife.

## Introduction

Heart failure has been a public health issue with an estimate of global prevalence over 37.7 million patients (1). Patients with diabetes and pre-diabetes have a higher risk of developing heart failure (2). It was found that the long-term hyperglycemia can cause damage to the myocardial structure and function (3).

Glycemic variability is one of the representative measures of glycemic control, such as fasting glucose (FG) variability and hemoglobin A1c (HbA1c) variability. It has begun to attract attention as a risk factor of increased adverse outcomes in the recent years. It was found that glycemic variability was associated with both the microvascular and macrovascular complications and mortality in diabetes (4, 5). In addition, it was found that the greater visit-to-visit variability of FG was associated with all-cause mortality risk, which is particularly noteworthy since the significance of the association between FG variability and mortality was greater in magnitude in non-diabetic population than diabetic population (6).

However, in community-based young population, over and above the effect of mean blood glucose, whether a higher level of glycemic variability has independent adverse effect on the left ventricular structure and function in midlife after multivariable adjustment is unclear. The current study aimed to determine the unestablished association of the greater visit-to-visit fasting glucose (FG) variability in young adulthood among the community population associated with cardiac function alteration and cardiac remodeling at midlife.

## Method

### Participants

The community-based cohort study of Coronary Artery Risk Development in Young Adult (CARDIA) is designed to recruit 5,115 healthy white and black adults from the four United States field sites (Birmingham, AL; Chicago, IL; Minneapolis, MN; and Oakland, CA). The participants accepted the baseline examination at age of 18–30 years during the period of 1985–1986 (Year 0), and follow-up examination at Year 2, 5, 7, 10, 15, 20, and 30. The participants with complete documented FG and echocardiography measurements were extracted from the 72% of the participants who had attended the examination at Year 25.

### Visit-to-Visit FG Variability

The participants were kept on fasting for at least 12 h and then the fasting samples were taken with the standardized protocols. The hexokinase UV method was used as an assay to measure FG at Year 0, and the hexokinase coupled to glucose-6-phophate dehydrogenase was used at Year 2, 10, 15, 20, and 25. A calibration study approach was used to standardize the FG values through the whole course of CAIDIA project (7).

### Echocardiography Measurements

At Year 25, the participants had accepted an echocardiographic evaluation by well-trained sonographers using Artida Ultrasonographic System of Toshiba Corporation (Tokyo, Japan) with a 1.8- to 4.2-MHz phase-array transducer. The whole procedure was following the guideline of American Society of Echocardiography. Then, the digital images acquired were re-read and interpreted by the professional physicians. The reproducibility was reported to be good.

The peak early diastolic velocity (E) and early peak diastolic septal mitral annular velocity (e') were obtained by tissue Doppler mode, and E/e' was calculated. Left ventricular mass index (LVMI) was obtained using left ventricular (LV) mass calculated by Devereux formulation indexed to body surface area calculated by Du Bois formula (Kilogram/meter square). The relative wall thickness defined as two times posterior wall thickness divided by LV diastolic diameter under MM mode. Left ventricular ejection fraction (LVEF) was derived from LV volumes from apical views. The longitudinal strain was measured using speckle tracking echocardiography with Advanced Cardiology package 2D wall motion tracking (version 3), which represented the percentage of maximal length change of a LV segment during systole relative to its end-diastole length. Left ventricular hypertrophy is defined as left ventricular mass > 115 g/m^2^ for men and >95 g/m^2^ for women. Concentric remodeling is defined as relative wall thickness > 0.42. Impaired relaxation is defined as e' < 7 cm/s, the increased filling pressure is defined as E/e' ≥ 15 alone or E/e' 13–15, and the left atrial volume index ≥ 34 ml/m^2^.

### Other Covariates

The CARDIA study had collected information of the healthy risk factors using self-reported questionnaires, such as age, sex, race, smoking status, drinking status, degree of education, and physical activity concerned here.

The body mass index (BMI) was calculated using measured height and weight (the formula is BMI = kg/m^2^). The blood pressure (BP) measurement is conducted using standardized protocols. The participants observing fast and free of heavy physical activity for at least 12 h, were asked to sit quietly for 5 min before three times of measurements at a 1-min intervals were taken. We used the mean value of the second and third reading here. At Year 0–15, Hawksley (Lancing, United Kingdom) random zero sphygmomanometer was used while at Year 20–25, an automated BP measurement monitor was used. Therefore, the calibration of BP measurement at Year 20–25 was performed (8). The level of plasma cholesterol was measured by enzymatic assays using fasting plasma samples.

### Statistical Analyses

Continuous variables were descripted with mean and SD and categorical variables with proportions. The visit-to-visit FG variability were presented by SD of mean FG (SD_FG_), coefficient of variation of the mean FG (CV_FG_), and average real variation of mean FG (ARV_FG_). To determine the association between visit-to-visit variability and LV structure and function, the multivariable-adjusted linear regression models were used for E/e' and e', respectively. An additional exploratory analysis of cardiac systolic function and structure was conducted. In these models, covariates of age, sex, race, education, BMI, smoking and drinking status, systolic and diastolic BP, total cholesterol, high-density lipoprotein cholesterol (collected at Year 25), anti-hypertensive, anti-diabetic and lipid-lowing medication use history during the whole course of CARDIA study, and weight mean FG were adjusted. A sensitivity analysis of the association between visit-to-visit FG variability and LV structure and function in a subset of people with no history of antidiabetic drugs was conducted. The dependent variables were set as binary variables according to the 2016 American Society of Echocardiography/European Association of Cardiovascular Imaging guideline (9), and another sensitivity analysis of the association between visit-to-visit FG variability and the presence of adverse LV subclinical diastolic dysfunction was done. A two-tailed *P* < 0.05 was considered to indicate statistically significant. An analysis was performed using SPSS (version 25) (SPSS Inc., Chicago, IL, USA).

## Results

### Participants Characteristics

Of the 5,115 participants, 2,416 participants who did not attend the Year 25 echocardiographic examination were excluded. In addition 8 participants who were neither black nor white and a total of 91 participants with any missing covariates of fasting blood glucose (*n* = 54), BMI (*n* = 16), blood pressure (*n* = 9), and blood lipids (*n* = 12) were excluded from the study. The remaining 2,600 participants were included in the study.

In total 2,600 participants appeared for the examination at Year 0 and their mean (SD) age was 24.9 (3.6) years. There were 57.3% women and 46.7% African American. The mean (SD) BMI of the individuals at Year 0 was 23.9 (4.3). At Year 0, 25.7% of the participants were smokers and 87.3% were drinkers. At Year 25, the mean (SD) BMI was 29.3 (6.3), and 16.6% of the participants were smokers and 78.7% were drinkers. The mean (SD) of FG was 98.5 (28.0) mg/dL. Other baseline characteristics are shown in Table 1. The associations of SD_FG_, CV_FG_, and ARV_FG_ with clinical characteristics are presented in Supplementary Tables 1, 2. The BMI, systolic blood pressure (SBP), diastolic blood pressure (DBP), and high-density lipoprotein (HDL) at Year 25 were associated with SD_FG_, CV_FG_, and ARV_FG_ (*P* < 0.001 for all). The results showed that men, black, current drinker, and non-smoker at Year 25, and the participants with antidiabetic, antihypertensive, and lipid-lowering medication use were more likely to have a higher SD_FG_, CV_FG_, and ARV_FG_ (*P* < 0.001 for all).

**Table 1:** Participants characteristics of study cohort (*n* = 2,601).

**Characteristics**	**Mean (SD)** **at year 0**	**Mean (SD)** **at year 25**
Age (years)	24.9 (3.6)	49.9 (3.6)
Women (%)	57.3	57.3
Black (%)	46.7	46.7
Education (years)	–	14.0 (2.0)
BMI (kg/m^2^)	23.9 (4.3)	29.3 (6.3)
Current smoker (%)	25.7	16.6
Current drinker (%)	87.3	78.7
SBP (mmHg)	109.5 (10.6)	118.1 (15.1)
DBP (mmHg)	68.0 (9.3)	73.5 (10.7)
FG (mg/dL)	81.5 (10.0)	98.5 (28.0)
TC (mg/dL)	176.5 (32.6)	192.5 (36.3)
HDL-c (mg/dL)	53.5 (12.6)	58.7 (18.1)
Antihypertensive medication use (%)	1.8	24.1
Antidiabetic medication use (%)	0.6	6.2
Lipid-lowering medication use (%)	2.2	14.4
Visit-to-visit SD_FG_ mg/dL	–	9.7 (12.0)
Visit-to-visit CV_FG_ mg/dL	–	10.0 (8.2)
Visit-to-visit ARV_FG_ mg/dL	–	10.4 (11.3)
**Cardiac structure**		
LVMI	–	84.3 (21.1)
Relative wall thickness	–	0.5 (0.1)
**Left ventricular diastolic function**		
e'	–	9.4 (2.4)
E/e'	–	8.9 (2.8)
**Left ventricular systolic function**		
EF	–	69.7 (7.9)
Longitudinal strain	–	−15.1 (2.4)

*Data are presented as mean (SD) or percentage (%) as appropriate*.

*BMI, body mass index; SBP, systolic blood pressure; DBP, diastolic blood pressure; FG, fasting glucose; TC, total cholesterol; HDL-c, high-density lipoprotein cholesterol; e', Tissue doppler septal E-wave prime velocity; E/e', the ratio between early mitral inflow velocity and mitral annular early diastolic velocity; EF, Left ventricular ejection fraction; LVMI, LV Mass Indexed to Body Surface Area; N/A, not applicable*.

### Multivariate Linear Regression Analysis

**Figure 1:**
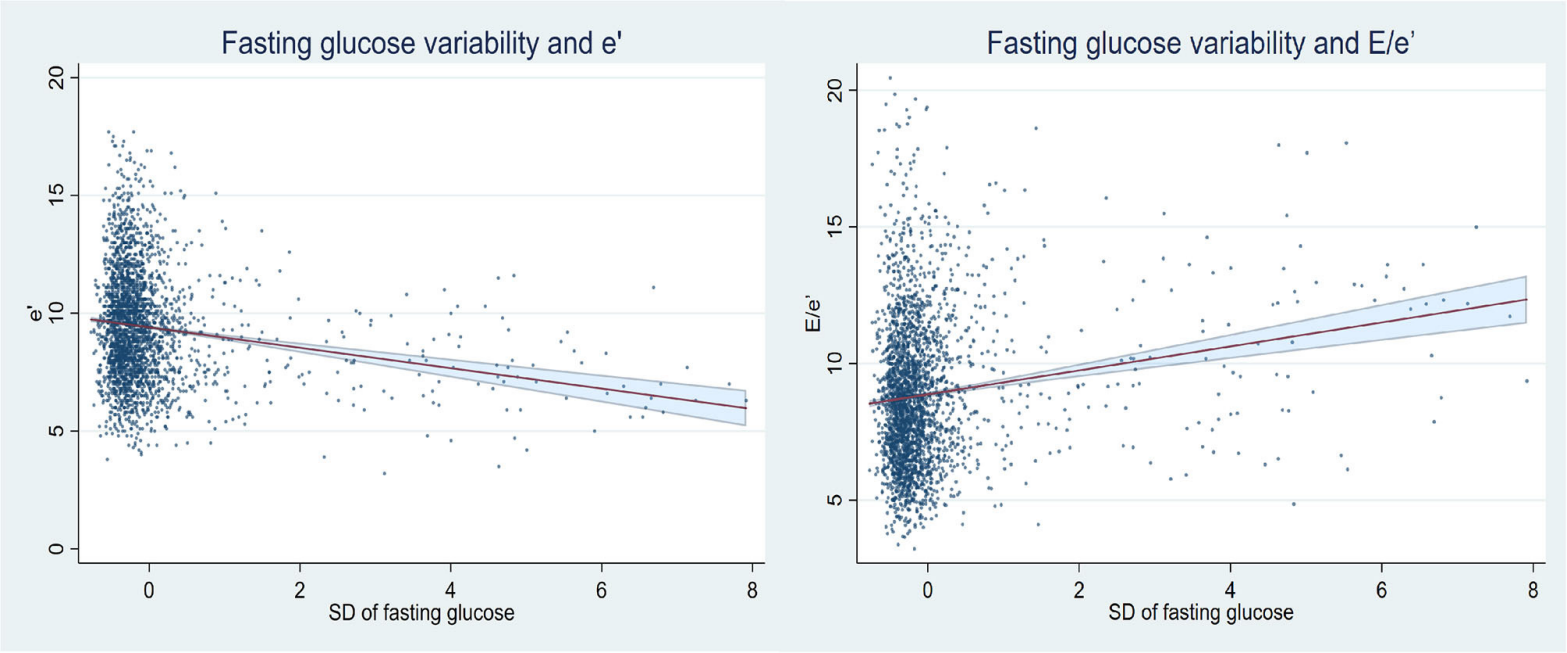
Linear plots and 95% CIs for the association between FG variability (SD) and outcomes of E/e' and e'.

After multivariable adjustment, the association with LV structure and function at Year 25 is presented in Table 2, adjusted β (SE) of other clinical variables is presented in Supplementary Table 5. The higher SD_FG_ is associated with lower e' (β [SE], −0.214 [0.080], *P* < 0.01) and higher E/e' (β [SE], 0.307 [0.094], *P* < 0.01), and higher CV_FG_ is also associated with lower e' (β [SE], −0.141 [0.066], *P* < 0.05) and higher E/e' (β [SE], 0.204 [0.078], *P* < 0.01). An exploratory analysis showed that the higher ARV_FG_ is associated with the lower higher E/e' (β [SE], 0.178 [0.085], *P* < 0.05) and higher LVMI (β [SE], 1.240 [0.618], *P* < 0.05). The linear plots and 95% *CI*s for the association between FG variability (SD) and outcomes of E/e' and e' are presented in Figure 1. None of the variables of FG variability were found to be significantly associated with LV systolic function.

**Table 2:** Association between visit-to-visit FG variability and cardiac structure and function at year 25 (the CARDIA Study).

	**SD**_**FG**_ **(per 1 SD increased)**		**CV**_**FG**_ **(per 1 SD increased)**		**ARV**_**FG**_ **(per 1 SD increased)**	
	**Unadjusted β (SE)**	**Adjusted β (SE)**	**Unadjusted β (SE)**	**Adjusted β (SE)**	**Unadjusted β (SE)**	**Adjusted β (SE)**
**Cardiac structure**						
LVMI	2.982 (0.410)	0.911 (0.689)	2.754 (0.411)	0.445 (0.570)	3.070 (0.410)	1.240 (0.618)^*^
RWT	0.005 (0.002)	−0.002 (0.003)	0.005 (0.002)	−0.002 (0.002)	0.005 (0.002)	−0.002 (0.003)
**Systolic function**						
LVEF	−0.318 (0.154)	−0.353 (0.283)	−0.262 (0.154)	−0.156 (0.234)	−0.288 (0.154)	−0.276 (0.253)
GLS	0.449(0.047)	0.104 (0.080)	0.430 (0.047)	0.038 (0.066)	0.415 (0.047)	0.083 (0.072)
**Diastolic function**						
e'	−0.402 (0.046)	−0.214 (0.080)^†^	−0.401 (0.046)	−0.141 (0.066)^*^	−0.365 (0.046)	−0.127 (0.072)
E/e'	0.430 (0.054)	0.307 (0.094)^†^	0.432 (0.054)	0.204 (0.078)^†^	0.395 (0.054)	0.178 (0.085)^*^

*FG, fasting glucose; SD, standard deviation; CV, coefficient of variation; ARV, average real variability of mean FG; LVMI, LV mass index; RWT, Relative wall thickness; LVEF, left-ventricular ejection fraction; GLS, global longitudinal strain; e', early peak diastolic mitral annular velocity; E/e', mitral inflow velocity to early diastolic mitral annular velocity*.

*Models were adjusted for year 25 covariates: age, sex, race, level of education, body mass index, smoking status, drinking status, systolic blood pressure, diastolic blood pressure, total cholesterol level, high-density lipoprotein cholesterol level, antihypertensive, antidiabetic and lipid-lowing medication use (year 0–25), and weighted mean fasting glucose*.

* *P < 0.05*.

† *P < 0.01*.

### Sensitivity Analysis

The sensitivity analysis of association between visit-to-visit FG variability and LV structure and function at Year 25 in the subset of participants who had no antidiabetics drug (*n* = 2,438) use history was done (Supplementary Table 3), and the result of this subset showed that visit-to-visit FG variability had been consistently associated with e' (SD_FG_, β [SE], −0.165 [0.057], *P* < 0.01; CV_FG_, β [SE], −0.102 [0.051], *P* < 0.05) and E/e' (SD_FG_, β [SE], 0.209 [0.066], *P* < 0.01; CV_FG_, β [SE], 0.133 [0.059], *P* < 0.05). In the sensitivity analysis of association between visit-to-visit FG variability and the presence of adverse subclinical diastolic dysfunction in total study population (*n* = 2,600), SD_FG_ was associated with impaired relaxation (OR [95% CI], 1.22 [1.02, 1.45], *P* < 0.05) (Supplementary Table 4).

## Discussion

Based on a large community-based cohort of young biracial adults who followed-up for 25 years, this study found that the greater visit-to-visit FG variability in young adulthood is associated with higher E/e' and larger LVMI at midlife. In a subset of participants with no antidiabetics drug use history, the main result remains consistent, which implicated that the FG variability in early life is associated with the occurrence of subclinical diastolic dysfunction and cardiac remodeling in later age.

In this study, SD_FG_ and CV_FG_ can be seen as the metrics of overall variability and ARV_FG_ can be seen as the metrics of variability between consecutive visits. All of the three variables of SD, CV, and ARV reflect the variability of FG. Although the result of association between ARV_FG_ and e' is statistically insignificant, it cannot not negate the positive results of this study. Because to demonstrate the association does not require three positive *P*-values, the results from any of the three methods are authentic (10). The CARDIA was designed to be a cohort of young community-based population, comparing with other studies or cohorts, the number of positive events are not that large. Therefore, based on a sample from CARDIA, even the result has only indicated a population-level change in e' and E/e', it could have implications for a prognostic value of adverse outcome from a public health perspective (11–13).

A previous study had revealed the association between visit-to-visit FG variability of 4.7 years and LV structure and systolic function change in patients with type 2 diabetes (14). But its sample size was small, and its follow-up interval was not equal. Our study expanded the study population from small-scale type 2 diabetes patients to larger-sized community cohort, as CARDIA provides an ideal setting that can explore the glycemic variability of people from young adulthood to middle-age in natural state. The current study is the first to reveal the association between higher long-term visit-to-visit FG variability and preclinical change of LV diastolic function as well as adverse change in LVMI in young community-based population. It is noteworthy as the decreased LV diastolic function and cardiac remodeling in the subclinical states may increase the risk of heart failure and other cardiovascular diseases (15–18). Moreover, a large number of studies have shown that the glycemic variability had been a risk factor for adverse outcome in various metabolic diseases. For example, it is associated with increased mortality in the diabetes patients who are older than 70 years of age (19), and cognitive decline among the elderly population with or without diabetes (20, 21), etc. Moreover, among the young individuals in CARDIA study, visit-to-visit glycemic variability during young adulthood was also associated with increased incident of diabetes, cardiovascular disease, and mortality (22), a decline of cognitive function (23). Our previous research found that FG variability was associated with hippocampal structural damage (24). This study found that visit-to-visit glycemic variability during young adulthood among the community-based population was related to changes in the middle-aged cardiac structure and diastolic function, which provided increased evidence that glycemic variability could have an adverse effect on long-term outcome. Therefore, the glycemic variability should be brought to our attention in the future clinical practice.

The findings have indicated that the higher glycemic variability could cause damage to both microvascular and macrovascular. The *in-vitro* studies showed that NADPH oxidase played vital role in inducing superoxide production when blood glucose level fluctuated (25), and the role of oxidative stress by inhibiting the protein kinase B (AKT) pathway had been found involved in the progress of heart tissue fibrosis caused by the glycemic variability (26). In studies of human subjects, oscillating glucose had been found to be more damaging to the vascular endothelial function than stables the constant high glucose, in which progressive oxidative stress plays a key role (27). A study in FG normal control individuals and in type 1 diabetes confirmed the effect of the way of recovering from hypoglycemia, since the progress induces endothelial dysfunction, oxidative stress, and inflammation, the deleterious effects of the recovering progress could be worsen when obtained reaching hyperglycemia comparing with reaching normoglycemia (28). This needs to be further verified that the higher glycemic variability may have greater effect on the cardiovascular risk.

The strengths of the study include a prospective design, a long-term of 25 years follow-up from young adulthood to middle age, a large community-based cohort with samples free of diabetes at baseline, standardized collection protocols of data, and equal follow-up intervals, good retention, and quality control. The limitations of the study are as follow, first, the CARDIA study did not arrange follow-up test for HbA1c, of which if available, could make the conclusion more reliable. Second, the follow-up interval of the CARDIA study was no shorter than 5 years, hence the calculated index of variability was relatively coarse, which may cover up the glucose fluctuation that could not be assessed. But as the conservative data had also obtained a positive result, we can infer that our conclusion is reliable. Third, as it is an observational study, there is the possibility of residual confounding from omitting confounders, and the association might be due to the reverse causality.

In conclusion, we found that the greater visit-to-visit FG variability in young adulthood is associated with higher E/e' at midlife, greater SD_FG_ and CV_FG_ are associated with lower e' in midlife. This finding may be valuable for evaluating the potential risk for diastolic dysfunction. The manner of glucose lowering may be as important as the degree of glucose lowering.

## Data Availability Statement

Publicly available datasets were analyzed in this study. This data can be found here: https://www.cardia.dopm.uab.edu/invitation-to-new-investigators.

## Ethics Statement

Written informed consent was obtained from the individual(s), and minor(s)' legal guardian/next of kin, for the publication of any potentially identifiable images or data included in this article.

## Author Contributions

ZX, PX, XZhu, and XL: research idea and study design. XZhu and Z-cC: data acquisition. JL, XZhu, and YL: data analysis/interpretation. ZX, PX, and SZ: statistical analysis. ZX, PX, HZ, and ML: manuscript drafting. XL is the guarantor of this work and, as such, had full access to all the data in the study and takes responsibility for the integrity of the data and the accuracy of the data analysis. All authors contributed important intellectual content during manuscript writing or revision, and read and approved the final manuscript.

## Funding

XL was supported by the National Natural Science Foundation of China (81600206 to XZhu; 81870195 to XL), and Natural Science Foundation of Guangdong Province (2016A030310140 to XZhu; 20160903 to XL). The funders had no role in study design, data collection and analysis, decision to publish, or preparation of the manuscript. The CARDIA study was conducted and supported by the National Heart, Lung, and Blood Institute (NHLBI) in collaboration with the University of Alabama at Birmingham (HHSN268201300025C and HHSN268 201300026C), Northwestern University (HHSN268201300027C), University of Minnesota (HHSN268201300028C), Kaiser Foundation Research Institute (HHSN268201300029C), and Johns Hopkins University School of Medicine (HHSN268200900041C). The CARDIA was also partially supported by the Intramural Research Program of the National Institute on Aging (NIA) and an intra-agency agreement between NIA and NHLBI (AG0005). This article has been reviewed by CARDIA for scientific content.

## Conflict of Interest

The authors declare that the research was conducted in the absence of any commercial or financial relationships that could be construed as a potential conflict of interest.

## Publisher's Note

All claims expressed in this article are solely those of the authors and do not necessarily represent those of their affiliated organizations, or those of the publisher, the editors and the reviewers. Any product that may be evaluated in this article, or claim that may be made by its manufacturer, is not guaranteed or endorsed by the publisher.

## References

[1] Vos T Flaxman AD Naghavi M Lozano R Michaud C Ezzati M. Years lived with disability (YLDs) for 1160 sequelae of 289 diseases and injuries 1990-2010: a systematic analysis for the global burden of disease study 2010. Lancet. (2012) 380:2163–96. 10.1016/S0140-6736(12)61729-2 23245607 PMC6350784

[2] Lehrke M Marx N. Diabetes mellitus and heart failure. Am J Cardiol. (2017) 120:S37–47. 10.1016/j.amjcard.2017.05.014 28606342

[3] Jørgensen PG Jensen MT Mogelvang R Fritz-Hansen T Galatius S Biering-Sørensen T. Impact of type 2 diabetes and duration of type 2 diabetes on cardiac structure and function. Int J Cardiol. (2016) 221:114–21. 10.1016/j.ijcard.2016.07.083 27423078

[4] Gorst C Kwok CS Aslam S Buchan I Kontopantelis E Myint PK. Long-term glycemic variability and risk of adverse outcomes: a systematic review and meta-analysis. Diabetes Care. (2015) 38:2354–69. 10.2337/dc15-1188 26604281

[5] Ceriello A Monnier L Owens D. Glycaemic variability in diabetes: clinical and therapeutic implications. Lancet Diabetes Endocrinol. (2019) 7:221–30. 10.1016/S2213-8587(18)30136-0 30115599

[6] Echouffo-Tcheugui JB Zhao S Brock G Matsouaka RA Kline D Joseph JJ. Visit-to-visit glycemic variability and risks of cardiovascular events and all-cause mortality: the ALLHAT study. Diabetes Care. (2019) 42:486–93. 10.2337/dc18-1430 30659073 PMC6463548

[7] Park K Gross M Lee DH Holvoet P Himes JH Shikany JM. Oxidative stress and insulin resistance: the coronary artery risk development in young adults study. Diabetes Care. (2009) 32:1302–7. 10.2337/dc09-0259 19389821 PMC2699736

[8] Yano Y Reis JP Levine DA Bryan RN Viera AJ Shimbo D. Visit-to-visit blood pressure variability in young adulthood and hippocampal volume and integrity at middle age: the CARDIA study (coronary artery risk development in young adults). Hypertension. (2017) 70:1091–8. 10.1161/HYPERTENSIONAHA.117.10144 28993449 PMC5680098

[9] Nagueh SF Smiseth OA Appleton CP Byrd BF 3rd Dokainish H Edvardsen T. Recommendations for the evaluation of left ventricular diastolic function by echocardiography: an update from the American Society of Echocardiography and the European Association of Cardiovascular Imaging. Eur Heart J Cardiovasc Imaging. (2016) 17:1321–60. 10.1093/ehjci/jew082 27422899

[10] Levitan EB Kaciroti N Oparil S Julius S Muntner P. Relationships between metrics of visit-to-visit variability of blood pressure. J Hum Hypertens. (2013) 27:589–93. 10.1038/jhh.2013.19 23535987

[11] Armstrong AC Jacobs DR Jr. Gidding SS Colangelo LA Gjesdal O. Framingham score and LV mass predict events in young adults: CARDIA study. Int J Cardiol. (2014) 172:350–5. 10.1016/j.ijcard.2014.01.003 24507735 PMC4068332

[12] Nwabuo CC Moreira HT Vasconcellos HD Mewton N Opdahl A Ogunyankin KO. Left ventricular global function index predicts incident heart failure and cardiovascular disease in young adults: the coronary artery risk development in young adults (CARDIA) study. Eur Heart J Cardiovasc Imaging. (2019) 20:533–40. 10.1093/ehjci/jey123 30247530 PMC6477648

[13] Nwabuo CC Yano Y Moreira HT Appiah D Vasconcellos HD Aghaji QN. Association between visit-to-visit blood pressure variability in early adulthood and myocardial structure and function in later life. JAMA Cardiol. (2020) 5:795–801. 10.1001/jamacardio.2020.0799 32293640 PMC7160747

[14] Tang X Zhong J Zhang H Luo Y Liu X Peng L. Visit-to-visit fasting plasma glucose variability is an important risk factor for long-term changes in left cardiac structure and function in patients with type 2 diabetes. Cardiovasc Diabetol. (2019) 18:50. 10.1186/s12933-019-0854-9 30992008 PMC6469221

[15] Velagaleti RS Gona P Pencina MJ Aragam J Wang TJ Levy D. Left ventricular hypertrophy patterns and incidence of heart failure with preserved versus reduced ejection fraction. Am J Cardiol. (2014) 113:117–22. 10.1016/j.amjcard.2013.09.028 24210333 PMC3881171

[16] Yeboah J Rodriguez CJ Stacey B Lima JA Liu S Carr JJ. Prognosis of individuals with asymptomatic left ventricular systolic dysfunction in the multi-ethnic study of atherosclerosis (MESA). Circulation. (2012) 126:2713–9. 10.1161/CIRCULATIONAHA.112.112201 23124035 PMC3533250

[17] Bluemke DA Kronmal RA Lima JA Liu K Olson J Burke GL. The relationship of left ventricular mass and geometry to incident cardiovascular events: the MESA (Multi-Ethnic Study of Atherosclerosis) study. J Am Coll Cardiol. (2008) 52:2148–55. 10.1016/j.jacc.2008.09.014 19095132 PMC2706368

[18] Armstrong AC Liu K Lewis CE Sidney S Colangelo LA Kishi S. Left atrial dimension and traditional cardiovascular risk factors predict 20-year clinical cardiovascular events in young healthy adults: the CARDIA study. Eur Heart J Cardiovasc Imaging. (2014) 15:893–9. 10.1093/ehjci/jeu018 24534011 PMC4215562

[19] Forbes A Murrells T Mulnier H Sinclair AJ. Mean HbA(1c), HbA(1c) variability, and mortality in people with diabetes aged 70 years and older: a retrospective cohort study. Lancet Diabetes Endocrinol. (2018) 6:476–86. 10.1016/S2213-8587(18)30048-2 29674135

[20] Tamura Y Kimbara Y Yamaoka T Sato K Tsuboi Y Kodera R. White matter hyperintensity in elderly patients with diabetes mellitus is associated with cognitive impairment, functional disability, and a high glycoalbumin/glycohemoglobin ratio. Front Aging Neurosci. (2017) 9:220. 10.3389/fnagi.2017.00220 28729834 PMC5498506

[21] Zhang T Su G Mi SH Yang HX Xin W Dai WL. Association between blood glucose variability and the characteristics of vulnerable plaque in elderly non-st segment elevation acute coronary syndrome patients. Int Heart J. (2019) 60:569–76. 10.1536/ihj.18-503 31019178

[22] Bancks MP Carson AP Lewis CE Gunderson EP Reis JP Schreiner PJ. Fasting glucose variability in young adulthood and incident diabetes, cardiovascular disease and all-cause mortality. Diabetologia. (2019) 62:1366–74. 10.1007/s00125-019-4901-6 31115643 PMC7235631

[23] Bancks MP Carnethon MR Jacobs DR Jr. Launer LJ Reis JP. Fasting glucose variability in young adulthood and cognitive function in middle age: the coronary artery risk development in young adults (CARDIA) study. Diabetes Care. (2018) 41:2579–85. 10.2337/dc18-1287 30305344 PMC6245206

[24] Xiong Z Li J Zhong X Zhang S Sun X Zhou H. Visit-to-visit fasting glucose variability in young adulthood and hippocampal integrity and volume at midlife. Diabetes Care. (2019) 42:2334–7. 10.2337/dc19-0834 31548243 PMC7364671

[25] Maeda M Hayashi T Mizuno N Hattori Y Kuzuya M. Intermittent high glucose implements stress-induced senescence in human vascular endothelial cells: role of superoxide production by NADPH oxidase. PLoS ONE. (2015) 10:e0123169. 10.1371/journal.pone.0123169 25879533 PMC4400006

[26] Ying C Liu T Ling H Cheng M Zhou X Wang S. Glucose variability aggravates cardiac fibrosis by altering AKT signalling path. Diab Vasc Dis Res. (2017) 14:327–35. 10.1177/1479164117698917 28301953

[27] Ceriello A Esposito K Piconi L Ihnat MA Thorpe JE Testa R. Oscillating glucose is more deleterious to endothelial function and oxidative stress than mean glucose in normal and type 2 diabetic patients. Diabetes. (2008) 57:1349–54. 10.2337/db08-0063 18299315

[28] Ceriello A Novials A Ortega E La Sala L Pujadas G Testa R. Evidence that hyperglycemia after recovery from hypoglycemia worsens endothelial function and increases oxidative stress and inflammation in healthy control subjects and subjects with type 1 diabetes. Diabetes. (2012) 61:2993–7. 10.2337/db12-0224 22891214 PMC3478543

