# Supplementary material for: Visit-to-Visit Fasting Glucose Variability in Young Adulthood and Cardiac Structure and Function at Midlife: The CARDIA Study
Source: Front Cardiovasc Med. 2021 Sep 16;8:687054. doi: 10.3389/fcvm.2021.687054 (PMC8481606; doi:10.3389/fcvm.2021.687054)
Supplement: Supplementary file 1 [file Table_1.DOCX]

eTable 1. Correlations between FG variability measurement and clinical characteristics (n=2600)

| **Variables** | **SD_FG_, mg/dL** | **P value** | **CV_FG_, mg/dL** | **P value** | **ARV_FG_, mg/dL** | **P value** |
| --- | --- | --- | --- | --- | --- | --- |
| Age at Y_25_, years | 0.993 | <0.001 | -0.005 | 0.779 | 0.009 | 0.646 |
| Body mass index at Y_25_, kg/m^2^ | 0.130 | <0.001 | 0.147 | <0.001 | 0.103 | <0.001 |
| Systolic blood pressure at Y_25_, mmHg | 0.121 | <0.001 | 0.141 | <0.001 | 0.119 | <0.001 |
| Diastolic blood pressure at Y_25_, mmHg | 0.091 | <0.001 | 0.121 | <0.001 | 0.077 | <0.001 |
| Total cholesterol at Y_25_, mg/dL | -0.026 | 0.183 | -0.030 | 0.128 | -0.032 | 0.104 |
| High-density lipoprotein at Y_25_, mg/dL | -0.144 | <0.001 | -0.137 | <0.001 | -0.118 | <0.001 |
| Pearson’s correlation coefficients are shown. FG variability measurements were calculated based on 5 office FGs measured from the Y0 to Y25 examinations. Statistical significance was defined as P <0.05. | | | | | | |

eTable 2. Differences in visit-to-visit FG variability by clinical characteristics (n=2600)

| **Characteristics** | **SD_FG_, mg/dL** | **P value** | **CV_FG_, mg/dL** | **P value** | **ARV_FG_, mg/dL** | **P value** |
| --- | --- | --- | --- | --- | --- | --- |
| **Sex** | | | | | | |
| Men, n=1110 | 10.6 (13.4) | 0.001 | 10.5 (8.9) | 0.010 | 11.1 (11.5) | 0.005 |
| Women, n=1490 | 9.0 (10.7) |  | 9.6 (7.6) |  | 9.8 (11.0) |  |
| **Race** | | | | | | |
| White, n=1387 | 8.2 (9.3) | <0.001 | 8.8 (6.5) | <0.001 | 12.0 (13.9) | <0.001 |
| Black, n=1213 | 11.4(814.2) |  | 11.4 (9.6) |  | 9.0 (8.3) |  |
| **Current smoker at Y_25_** | | | | | | |
| Yes, n=432 | 11.1 (13.8) | 0.021 | 11.3 (9.4) | 0.001 | 12.2 (14.4) | 0.003 |
| No, n=2168 | 9.4 (11.6) |  | 9.7 (7.9) |  | 10.0 (10.5) |  |
| **Current drinker at Y_25_** | | | | | | |
| Yes, n=2046 | 9.4 (10.8) | 0.021 | 9.8 (7.6) | 0.030 | 10.0 (9.7) | 0.019 |
| No, n=554 | 11.0 (15.4) |  | 10.8 (10.0) |  | 11.7 (15.8) |  |
| **Antidiabetic medication use at Y_25_** | | | | | | |
| Yes, n=162 | 39.0 (27.9) | <0.001 | 29.9 (15.9) | <0.001 | 36.1 (28.5) | <0.001 |
| No, n=2438 | 7.8 (6.4) |  | 8.7 (5.2) |  | 8.7 (5.9) |  |
| **Antihypertensive medication use at Y_25_** | | | | | | |
| Yes, n=626 | 13.9 (17.5) | <0.001 | 13.1 (11.3) | <0.001 | 14.2 (17.4) | <0.001 |
| No, n=1974 | 8.4 (9.2) |  | 9.0 (6.6) |  | 9.2 (8.1) |  |
| **Lipid-lowering medication use from at Y_25_** | | | | | | |
| Yes, n=274 | 16.6 (20.9) | <0.001 | 14.7 (13.5) | <0.001 | 16.5 (20.8) | <0.001 |
| No, n=2226 | 8.6 (9.2) |  | 9.2 (6.7) |  | 9.4 (8.3) |  |
| P values were obtained by one-way analysis of variance. Statistical significance was defined as P <0.05. | | | | | | |

eTable 3. Sensitivity analysis of association between visit-to-visit FG variability and cardiac structure and function at Year 25 in subset of participates who had no antidiabetics drug use history (the CARDIA Study) (n=2600)

| **Characteristics** | **adjusted β(SE) of SD_FG_** | **adjusted β(SE) of CV_FG_** | **adjusted β(SE) of ARV_FG_** |
| --- | --- | --- | --- |
| **Cardiac structure** | | | |
| LVMI | 1.438(0.476)† | 1.056(0.428)* | 2.037(0.444)‡ |
| RWT | 0.002(0.002) | 0.001(0.002) | 0.001(0.002) |
| **Systolic function** | | | |
| LVEF | -0.205(0.194) | -0.154(0.175) | -0.257(0.182) |
| GLS | 0.111(0.056)‡ | 0.058(0.051) | 0.126(0.053)* |
| **Diastolic function** | | | |
| e’ | -0.165(0.057)† | -0.102(0.051)* | -0.092 (0.053) |
| E/e’ | 0.209(0.066)† | 0.133(0.059)* | 0.117 (0.062) |

Abbreviations: FG: fasting glucose; SD: standard deviation; CV: coefficient of variation; ARV, average real variability of mean FG; LVMI, LV mass index; RWT: Relative wall thickness; LVEF, left-ventricular ejection fraction; GLS, global longitudinal strain; e’, early peak diastolic mitral annular velocity; E/e’, mitral inflow velocity to early diastolic mitral annular velocity;

Models were adjusted for year 25 covariates: age, sex, race, level of education, body mass index, smoking status, drinking status, systolic blood pressure, diastolic blood pressure, total cholesterol level, high-density lipoprotein cholesterol level, antihypertensive, antidiabetic and lipid-lowing medication use (year 0 to year 25), and weighted mean fasting glucose.

*:P<0.05, †:P<0.01, ‡:P<0.001.

eTable 4. Adjusted ORs and 95% CI for the presence of adverse left ventricular structural outcomes and systolic and diastolic dysfunction at Year 25 according to the variables of variability (The CARDIA Study). (n=2600)

| **Characteristics** | **OR (95%CI) of SD_FG_** | **OR (95%CI) of CV_FG_** | **OR (95%CI) of ARV_FG_** |
| --- | --- | --- | --- |
| **Cardiac structure** | | | |
| Left ventricular hypertrophy* | 1.08(0.92,1.27) | 1.01(0.87,1.17) | 1.05(0.87,1.25) |
| Concentric remodeling† | 0.95(0.80,1.09) | 0.97(0.85,1.10) | 0.98(0.84,1.14) |
| **Systolic function** | | | |
| Ejection fraction <55% | 1.15(0.90,1.47) | 1.18(0.92,1.27) | 1.23(0.94,1.61) |
| Longitudinal strain >90th | 1.07(0.91,1.28) | 1.04(0.89,1.22) | 1.05(0.87,1.26) |
| **Diastolic function** | | | |
| Impaired relaxation‡ | 1.22(1.02,1.45) ¶ | 1.13(0.98,1.31) | 1.07(0.91,1.26) |
| Increased filling pressure§ | 0.89(0.75,1.07) | 0.95(0.80,1.12) | 0.94 (0.77,1.15) |

Abbreviations: FG: fasting glucose; SD: standard deviation; CV: coefficient of variation; ARV, average real variability of mean FG; * Left ventricular mass >115 g/m^2^ for men and >95 g/m^2^ for women. † Relative wall thickness >0.42. ‡ e´ <7 cm/s. § E/e´ ≥15 alone or E/e´ 13-15 and left atrial volume index ≥ 34 ml/m.

Models were adjusted for year 25 covariates: age, sex, race, level of education, body mass index, smoking status, drinking status, systolic blood pressure, diastolic blood pressure, total cholesterol level, high-density lipoprotein cholesterol level, antihypertensive, antidiabetic and lipid-lowing medication use (year 0 to year 25), and weighted mean fasting glucose.

¶:P<0.05.

eTable 5. Adjusted β(SE) for cardiac structure and function at Year 25 according to SD of FG and other clinical variables (The CARDIA Study) (n=2600).

| **Variables** | **LVMI** | **RWT** | **LVEF** | **GLS** | **e’** | **E/e’** |
| --- | --- | --- | --- | --- | --- | --- |
| Age | 0.297(0.116)* | 0.001(0.001)† | 0.115(0.047)* | 0.031(0.013)* | -0.111(0.013)‡ | 0.084(0.016)‡ |
| Gender | -11.958(0.861)‡ | -0.008(0.004)* | 2.172(0.353)* | 0.697(0.100)‡ | 0.153(0.100) | 0. 781(0.118)‡ |
| Race | -0.965(0.904) | -0.024(0.004)‡ | -0.093(0.371) | -0.475(0.105)‡ | 0.032(0.105) | -0.246(0.123)* |
| Level of education | -0.603(0.224)† | -0.001(0.001) | 0.130(0.092) | -0.016(0.026) | 0.058(0.026)* | -0.072(0.031)* |
| BMI | 0.418( 0.072)‡ | <0.001(<0.001) | -0.012 (0.029) | 0.032(0.008)‡ | -0.022(0.008)† | 0.047(0.010)‡ |
| smoking status | 2.376(0.531)‡ | 0.005(0.002)* | -0.100(0.218) | 0.176(0.062)† | -0.154(0.062)* | 0.167(0.073)* |
| drinking status | 0.734(0.478) | 0.002(0.002) | 0.171(0.196) | -0.074(0.056) | 0.146(0.055)† | -0.215(0.065)† |
| SBP | 0.354(0.047)‡ | <0.001(<0.001) | 0.030(0.019) | -0.010(0.006) | -0.007(0.005) | 0.049(0.006)‡ |
| DBP | -0.226(0.068)† | <0.001(<0.001) | -0.045(0.028) | 0.051(0.008)‡ | -0.029(0.008)‡ | -0.029(0.009)† |
| TC | -0.035(0.011)† | <0.001(<0.001) | -0.005(0.004) | 0.001(0.001) | -0.001(0.001) | -0.012(0.001) |
| HDL | 0.065(0.025)† | 0.000(0.000)† | -0.010(0.010) | -0.012(0.003)‡ | 0.009(0.003)† | -0.008(0.003)* |
| Antihypertensive medication | 2.023(0.494)‡ | 0.003(0.002) | 0.439(0.203)* | 0.112(0.058) | -0.141(0.057)* | 0.129(0.068) |
| Antidiabetic medication | -2.743(1.065)* | -0.006(0.004) | -0.353(0.436) | 0.200(0.124) | 0.208(0.123) | -0.020(0.145) |
| Lipid-lowing medication | 0.257(0.599) | 0.005(0.002)* | 0. 208(0.245) | -0.049(0.070) | -0.069(0.069) | 0.082(0.082) |
| Mean fasting glucose | 0.119(0.053)* | <0.001(<0.001) | 0.010 (0.022) | 0.011(0.006) | 0.003(0.006) | -0.005(0.007) |
| SD_FG_ | 0.911(0.689) | -0.002(0.003) | -0.353(0.283) | 0.104(0.080) | -0.214(0.080)† | 0.307(0.094)† |

Abbreviations: FG: fasting glucose; SD: standard deviation; LVMI, LV mass index; RWT: Relative wall thickness; LVEF: left-ventricular ejection fraction; GLS: global longitudinal strain; e’: early peak diastolic mitral annular velocity; E/e’: mitral inflow velocity to early diastolic mitral annular velocity; BMI: body mass index; SBP: systolic blood pressure; DBP: diastolic blood pressure; TC: total cholesterol; HDL: high-density lipoprotein cholesterol.

Models were adjusted for year 25 covariates: age, gender, race, level of education, body mass index, smoking status, drinking status, systolic blood pressure, diastolic blood pressure, total cholesterol level, high-density lipoprotein cholesterol level, antihypertensive, antidiabetic and lipid-lowing medication use (year 0 to year 25), and weighted mean fasting glucose.

*:P<0.05, †:P<0.01, ‡:P<0.001.
